# In Vivo Production, Development and Storage of *Oscheius myriophila* (Nematoda: Rhabditida) in *Galleria mellonella* (Lepidoptera: Pyralidae)

**DOI:** 10.3390/microorganisms11102571

**Published:** 2023-10-16

**Authors:** Tania Marel Guadarrama-Avila, José Augusto Ramírez-Trujillo, Thania Gisel Rodríguez-Ocampo, Guadalupe Peña-Chora, Iván Arenas-Sosa, Víctor Manuel Hernández-Velázquez

**Affiliations:** 1 Laboratorio de Control Biológico, Centro de Investigación en Biotecnología, Universidad Autónoma del Estado de Morelos, Cuernavaca 62209, Mexico; tmga_1611@hotmail.com (T.M.G.-A.); augusto.ramirez@uaem.mx (J.A.R.-T.); thaniarodriguezocampo@gmail.com (T.G.R.-O.); 2 Laboratorio de Parasitología Vegetal, Centro de Investigaciones Biológicas, Universidad Autónoma del Estado de Morelos, Cuernavaca 62209, Mexico; penachora@uaem.edu.mx; 3 Departamento de Medicina Molecular y Bioprocesos, Instituto de Biotecnología, Universidad Nacional Autónoma de México, Coyoacán 04510, Mexico; ivan.arenas@ibt.unam.mx

**Keywords:** entomopathogenic nematodes, in vivo, storage

## Abstract

Entomopathogenic nematodes have been used in biological control for some time and are an alternative for the control of insect pests, but during their implementation, situations have arisen that can be improved. These vary with each species and include their production and storage. *Oscheius myriophila*, an entomopathogenic nematode (EPN), was monitored for its performance when produced in vivo, as well as its development using *Galleria mellonella* larvae, using the MC5-2014 strain isolated from soil samples in the municipality of Tepalcingo, Morelos, México. For a study with native strains of EPNs, a wide range of tests must be conducted because the required conditions can be very specific. In vivo production was quantified at initial infective juvenile (IJ) inocula of 50, 100 and 500, and we obtained the same production for the three inocula. The life cycle of the EPNs lasted 12 days, and two generations were observed in which adults were found at days 5 and 9. Both evaluations were performed at a temperature of 27 °C in *G. mellonella* larvae. In addition, the temperatures of 8, 12, 20 and 24 °C were evaluated for their storage, and we observed that the EPNs can be kept for at least 6 months, maintaining a survival rate of 58.67% and a good infective capacity at a temperature of 12 °C, remaining above 60%.

## 1. Introduction

Entomopathogenic nematodes (EPNs) are a promising alternative for the control of insect pests due to their wide range of hosts and active search method [1]. Although much information on EPNs has been generated since the 1980s, most of it focuses on the families Steinernematidae [2] and Heterorhabditidiae [3], which are associated with their symbiotic bacteria *Xenorhabdus* and *Photorhabdus*, respectively [4,5]. In addition to these families, species of EPNs belonging to the Rhabditidae family have been reported [6,7,8], and in turn, other species, for example, *Serratia*, have been reported to be symbiotic bacterial species [5,9].

Derived from EPN search and identification work, the Biological Control Laboratory of the Biotechnology Research Center of the Autonomous University of the State of Morelos (CEIB/UAEM) has EPNs that have not yet been reported in Mexico, one of which is *O. myriophila* [10], a species belonging to the Rhabditidae family.

For a study with native strains of EPNs, a wide range of tests must be conducted because the required conditions can be very specific; therefore, it is important to determine their development and the temperatures at which they are produced in greater volume and to preserve them for a longer period of time while maintaining their infectivity [11].

Carrying out propagations in vivo and determining the optimal conditions allowed us to perform an in-depth study. In vivo production is a simple process in which live host insects are used. This approach has various advantages, such as low start-up costs and little use of technology. This is an important production method because it allows us to obtain high-quality IJs. *G. mellonella* larvae are used as a substitute host [12], and are commercially produced in large quantities in several countries and susceptible to most nematode species [13].

In turn, *G. mellonella* is also used to observe the development of the EPN, learn more about it, and obtain information including its life cycle duration and the number of generations it could produce within the host [13,14,15]. In terms of the abovementioned families, the storage conditions for steinernematids involve temperatures that range from 8 to 15 °C, and they survive for 6 to 9 months; on the other hand, heterorhabdithids, when kept under the same conditions, survive for 3 to 4 months [16]. Therefore, knowing the requirements of each species is important in this field of study because they can vary considerably; currently, it is believed that it is best to use native species of EPNs in geographical areas where biocontrol is applied [17,18,19].

## 2. Materials and Methods

### 2.1. G. mellonella Production

*G. mellonella* was kept in the incubator at 30 ± 2 °C, and fed with a mixture that included rice flour, brewer’s yeast, wheat bran, glycerin and honey.

### 2.2. O. myriophila Strain MC5-2014 Propagation

At the bottom of 90 mm diameter × 15 mm depth Petri dishes, on a disk of medium-pore filter paper, 5 *G. mellonella* larvae were placed and 200 μL of suspension with EPNs was added for each larva; at the end of 5 days, the larvae were placed in white traps maintained at 27 °C with periods of daylight [20].

Once the EPNs reproduced and developed into new generations, they were deposited in 50 mL Corning culture flasks with 20 mL of sterile distilled water and kept refrigerated at 12 ± 1 °C to obtain the initial concentrated suspension [21].

### 2.3. Quantification of IJs

The method proposed by Woodring and Kaya [21] was used. For this, the initial suspension contained in the Corning flasks was homogenized. Then, 100 μL of suspension was obtained and deposited in a Petri dish, followed by counting the IJs with a stereoscopic microscope (Motic instruments, DM143, Hong Kong, China). This process was repeated five times, and the average counts were calculated to determine the inoculum of the EPN suspensions.

Using the formula of Woodring and Kaya [21], we obtained the required dilutions to use in each of the bioassays:

$$C=\frac{B\times A}{D}$$

where:

*A* = Volume of the concentrated suspension to be diluted (mL),

*B* = Number of nematodes in 1 mL diluted suspension (IJs/mL),

*C* = Final volume in milliliters of the new dilution (mL),

*D* = Desired nematode inoculum in the new dilution (IJs/mL) [21].

### 2.4. Bioassays

#### In Vivo Production

*G. mellonella* larvae were used, which were previously weighed; their weights were between 0.2 and 0.3 g.

The *G. mellonella* larvae were placed on medium-pore filter paper disks at the bottom of the Petri dishes (90 diameter × 15 mm depth). Then, we added different inoculum numbers of initial IJs, i.e., 50, 100 and 500 IJs.

Our experimental unit comprised 5 Petri dishes with *G. mellonella* larvae, adding the different inocula of 50, 100 and 500 IJs; thus, there were 3 treatments with 5 repetitions each. We evaluated the production obtained in each experimental unit, for which modified white traps were mounted once the larvae were completely dead, and a single trap was used for each repetition. Five larvae were in each group. All the larvae died from day 6 and were kept at a temperature of 27 °C. Throughout the experiment, the counts were made from day 8 of the larvae infection.

Monitoring was performed every 48 h by collecting the water from the white traps and counting the IJs produced during that time using a stereoscopic microscope (Motic instruments, DM143, China) [21].

### 2.5. Development of O. myriophila

To observe the development of the EPNs and confirm the presence of 2 or 3 generations, 14 Petri dishes with dimensions of 60 diameter × 15 mm depth were used with filter paper at the bottom, which was previously moistened with 400 µL of sterile distilled water. To each Petri dish, 5 larvae of *G. mellonella* and 500 IJs for each larva were added, with a total of 2500 IJs per Petri dish. The temperature was maintained at 27 °C, and monitoring started 8 h after placing the IJs. The progress was checked at 12 h and then every 24 h for the duration of the cycle, taking 5 larvae every 24 h during the days evaluated, and the different stages of *O. myriophila* were observed.

### 2.6. Fixation of EPNs

Five *G. mellonella* larvae exposed to the EPNs were taken; they were passed through 5% chlorine and sterile distilled water and later dissected and analyzed. The EPNs were observed with a stereoscopic microscope (Motic instruments, DM143, China). Later, they were fixed with 4% saline formalin at boiling point and individually mounted on a slide; the EPNs from each stage were taken [22].

### 2.7. Cleared of EPNs

To observe the EPNs in detail, they were evaluated after they were fixed using heat. A nematode was placed on a slide and different concentrations of glycerin and water were added in the following order: 1/20, 1/15, 1/10, 1/5 and 1/1. The different concentrations were added to the slides, which were placed on a heating plate.

When these processes were complete, the EPNs were observed under an optical microscope (Leica DM500, St. Gallen, Switzerland), and each stage was analyzed by measuring their length and width using Leica Application Suite, version 3.4.0 [23].

### 2.8. Storage of IJs of O. myriophila

Corning culture flasks (50 mL) were used, allowing the EPNs to receive oxygen. For each treatment (at temperatures of 8, 12, 24 and 27 °C), there were 15 flasks with a capacity of 50 mL, with 3000 IJs contained in 40 m water. We performed 3 repetitions since they were independent experiments. Subsequently, quantification was performed every 15 days to determine the survival percentage at different temperatures; thus, the mortality percentage was determined [21,24].

### 2.9. IJ Pathogenicity

To perform the pathogenicity bioassays, 4 flasks stored at various temperatures (8, 12, 20 and 27 °C) were taken at random, and they were quantified to obtain the average number of live and dead IJs. Once these data were obtained, a sixth or seventh instar *G. mellonella* larva was placed individually in a 60 diameter × 15 mm depth Petri dish with filter paper at the bottom, and a suspension of 250 μL of sterile distilled water with 10 IJs was added to each Petri dish with its respective *G. mellonella* larva.

Each experimental unit was made up of 10 *G. mellonella* larvae, with four repetitions for each temperature, and mortality was monitored at 48, 72, 96 and 120 h at 27 °C. The larvae were considered dead when a flaccid appearance and change in color appeared. Likewise, the presence of EPNs was verified with a stereoscopic microscope (Motic instruments, DM143, China).

### 2.10. Statistical Analysis

The obtained results in in vivo production were transformed with the square root method and analyzed with the statistical package SAS 9.1; ANOVA was performed with its respective Tukey test. Standard deviations of the means were calculated in Microsoft Excel Office 365.

The obtained data in bioassays of storage were transformed using arcsine and analyzed with an analysis of variance (ANOVA) and a Tukey multiple comparison of means at 0.05 probability using the R Commander statistical package 2.7-1 version. Standard deviations of the means were calculated in Microsoft Excel Office 365.

## 3. Results

### 3.1. In Vivo Production

When evaluating the production at three different initial inocula, different quantities were obtained and no significant differences were observed (Table 1).

### 3.2. Development of O. myriophila

**Figure 1:**
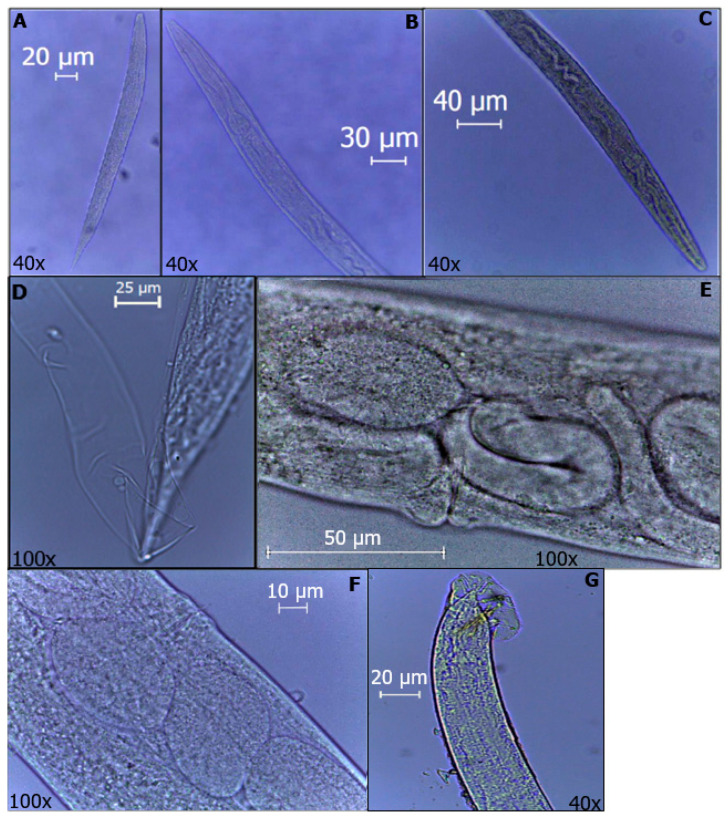
Developmental stages of *O. myriophila*. (**A**) J1. (**B**) J2. (**C**) J3. (**D**) J4. (**E**) Endotokia matricida of female. (**F**) Female with eggs inside. (**G**) Male rays and spicules are visible.

The development of the MC5-2014 strain belonging to the O. myriophila species was monitored at a temperature of 27 °C and an inoculum of 500 IJs per G. mellonella larva. From day 4, 100% mortality of the infected larvae was observed, and the following was discovered (Figure 1):

At 8 h of observation, J3 were found to be, on average, 526 µm long and 38 µm wide; the same was observed at 12 h; however, it was not until 48 h that there was a greater quantity within the analyzed larvae. At 96 h, J3 and J4 were approximately 510 µm long and 37 µm wide. At 120 h, both J4 and adults could be observed, i.e., the first generation, where the females were, on average, 1090 µm long and 78 µm wide; the matricidal endotochy process could be observed inside. Likewise, at 144 h, gravid females were observed that were 1320 µm long and 90 µm wide in addition to some J1, which were observed without visible organs inside and were, on average, 256 µm in length and 22 µm in width. Subsequently, at 168 h, J1, J2 and J3 were observed, which were 250 µm long and 22 µm wide, 386 µm long and 28 µm wide, and 582 µm long and 43 µm wide for each juvenile analyzed, respectively. 

Then, we observed mostly J4 and adults, and J4 showed average measurements of 615 µm in length and 40 µm in width. The females analyzed at 216 h were, on average, 906 µm in length and 65 µm in width, and matricidal endotochy was also observed in them. The males were approximately 850 µm in length and 56 µm in width.

To explain this in slightly more detail, we evaluated the measurements of different stages of the EPNs at different times using an optical microscope (Leica DM500, Switzerland) and the Leica Application Suite program, version 3.4.0. Their lengths and widths are shown in Table 2.

### 3.3. Storage IJs of O. myriophila

When analyzing the IJs stored for 15 days, a significant difference was observed. The IJs that were kept at 8 °C did not survive and exhibited a mortality rate of 100%; on the other hand, the IJs at temperatures of 12, 20 and 27 °C remained the same (*p* > 0.05), with survival rates of 93.33%, 88% and 85%, respectively.

No significant differences were observed in terms of survival, but at up to 120 days of analysis, survival decreased to 66.67% at 12 °C, 42.67% at 20 °C and 37.33% at 27 °C.

**Figure 2:**
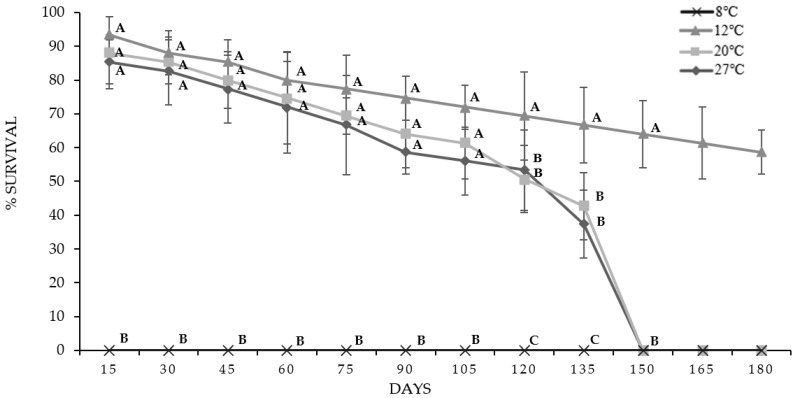
Survival percentages of IJs from *O. myriophila* analyzed for 180 days at four different temperatures; the mean ± SD is indicated. The same letters indicate that there are no significant differences (*p* < 0.05).

Likewise, at 150 days of storage, the IJs corresponding to temperatures of 20 and 27 °C decreased their survival percentage to 0%, unlike those preserved at 12 °C that maintained a survival rate of 58.67% at 180 days; this is illustrated in Figure 2.

The IJs at temperatures of 20 and 27 °C were kept for four and a half months; on the other hand, those kept at 12 °C maintained their survival for more than six months. Of note, the IJs stored at 8 °C had 100% mortality at the first evaluation, which was at day 15.

#### Pathogenicity of IJs

Pathogenicity was monitored after 30 days; there were no significant differences at the beginning (*p* > 0.05) because the infectivity percentages at this time were 97.5% at 12 and 20 °C and over 95% at 27 °C.

A significant difference in the evaluation could be observed at up to 90 days, and it was observed that the pathogenicity at 12 and 20 °C was significantly the same; on the other hand, a significant difference was observed between 12 and 27 °C. For the evaluation at 120 days, the pathogenicity was reduced to 20 and 27 °C and remained at 55% and 45%, respectively. Finally, at 180 days, the IJs preserved at 12 °C exhibited both survival and pathogenicity, which decreased at the same time (Table 3).

## 4. Discussion

In vivo propagation is used to produce EPNs on a laboratory scale and use them in various investigations, as well as to generate the necessary material for field tests [25]. White traps are needed to carry out this method, with good results when obtaining IJs.

According to Sáenz and Luque [26], the first days of emergence are those in which the greatest number of IJs are obtained, at up to 90% of the total production, and in our case, it was between days 6 and 14. The days with the highest production did not present significant differences, but this behavior was observed in the three inocula analyzed.

Likewise, other authors such as Realpe et al. [27] have carried out production studies with *G. mellonella* larvae using *Steinernema colombiense* and *Heterorhabditis bacteriophora*, and they obtained quantities of 86,250 and 78,750 IJs, respectively. Although these are higher figures than those obtained in the present study, averages of 165,000 IJs/larvae have also been recorded for different species, ranging between 30,000 and 240,000 IJs [28], thus approaching the reported yields.

Likewise, Boff and collaborators [29] mentioned that the decrease in IJs in in vivo propagation may be due to different factors, for example, age, size and even the susceptibility of the host insect used, in addition to different conditions, such as the environmental conditions and the inoculum used.

To complement the results obtained in in vivo propagation, it is also important to understand the development of the EPN over time by observing it, with the help of *G. mellonella* in this case. By observing the development of the EPN, it is known that the life cycle of the steinernematids is on average between 7 and 10 days and that of the heterorhabdithids is between 12 and 15 days [30], and they have between 1 and 2 generations throughout their development, which depend mainly on the availability of resources. Sáenz and Luque [26] monitored *Steinernema feltiae*, with an average cycle of 8 days. Additionally, the size of the adults was smaller in the second generation, which coincides with species of families other than the one used in this study [31].

To complement the results of the propagation and development of the nematode, its storage evaluation is also important. This is a strong point within the investigation, since there is little information about it, especially when we talk about specific species, such as the one addressed in this investigation. Understanding EPNs in detail is essential, in addition to knowing whether or not the evaluated conditions affect their pathogenicity. According to Kaya and Stock [16], the optimum storage temperatures for steinernematids is 4 to 15 °C for 6 to 9 months, and the heterorhabdithids under similar conditions can only be kept for 3 to 4 months; they also mention that inocula between 1000 and 2000 are ideal for storage and that most species are active at a temperature of 25 ± 2 °C.

In addition, there is information that extreme climates are detrimental to most EPN species, that is, temperatures greater than 30 °C or less than 9 °C, which vary depending on the geographical region where they are found [32].

Each species will withstand different temperatures since species belonging to the same family can persist at different temperatures; for example, *Steinernema glaseri* can withstand temperatures between 10 and 35 °C because it is a subtropical species. On the other hand, the *Steinernema carpocapsae* species persists best at temperatures between 5 and 15 °C [32].

Additionally, ecological studies of EPNs have shown that not only species isolates but also isolates within a species of EPNs differ in host range, infectivity, environmental stress tolerance, reproduction and host-finding ability [33,34,35,36,37,38,39].

The species we worked with was isolated from an area where the temperature ranges between 13 and 34 °C throughout the year, and it could not withstand the temperature of 8 °C at which it was evaluated and in which null survival was observed from the beginning of the storage experiment.

Therefore, temperature significantly affects storage because the survival and therefore also the infectivity of the IJs depend on it [40,41].

## Figures and Tables

**Table 1:** Number of IJs belonging to *O. myriophila* produced in *G. mellonella* larvae at different days with three different initial inocula of IJs at a temperature of 27 °C; the mean ± SD is shown. *p* > 0.05 (the same letters indicate no significant differences).

Temperature	Inoculum of IJs	IJs/Larvae Mean ± SD
	[50]	13,100 ± 4319 A
27 °C	[100]	10,849 ± 2716 A
	[500]	19,166 ± 7462 A

No significant differences were observed between each of the initial inocula used. The monitoring was performed for 38 days, which was when the corpse of the *G. mellonella* larva was exhausted.

**Table 2:** The results of the development of *O. myriophila*, where length and width measurements at the different stages of development were determined (mean ± SD) and the respective *n* in each case was observed (N/A = nonviable IJs).

Hours	Stage	Mean ± SD		*n*
		Length	Width	
8	J3	526.66 ± 20.54	38.33 ± 2.35	3
12	J3	535 ± 11.18	35.75 ± 3.76	4
24	N/A	N/A	N/A	N/A
48	J3	525 ± 20.61	33.83 ± 3.43	6
72	J3	510 ± 28.86	37 ± 3.16	7
96	J4	608 ± 30.22	42.6 ± 2.24	5
120	J4	610 ± 26.07	43.6 ± 1.95	5
	Female	1090 ± 48.98	78 ± 6.78	5
144	Female	1320 ± 52.38	90 ±3.16	5
	J1	256 ± 25.76	22 ± 1.89	5
168	J1	250 ± 18.97	22 ± 2.44	5
	J2	386 ± 64.37	28 ± 6.78	5
	J3	582 ± 38.67	43 ± 5.09	5
192	J4	615 ± 35.93	40.8 ± 3.43	6
	Female	850 ± 44.72	68 ± 2.44	5
216	Female	906 ± 33.82	65 ± 3.16	5
240	Female	990 ± 66.33	75 ± 4.47	5
	J1	240 ± 27.56	22 ± 2.44	5
264	J3	516.66 ± 23.57	35 ± 2.88	6
	J4	576 ± 22.44	56 ± 5.09	5
	Female	980 ± 24.49	80 ± 3.16	5
	Male	850 ± 54.77	56 ± 8.60	5
288	J4	620 ± 40	55 ± 3.16	5
	Female	1008.33 ± 53.35	83.33 ± 2.35	6

**Table 3:** Percentage rates of pathogenicity of IJs on 10 *G. mellonella* larvae evaluated over 180 days at different temperatures (*n* = 10). The values are shown as the mean ± SD. The same letters indicate that there are no significant differences (*p* < 0.05). (N/A = nonviable IJs).

*O. myriophila*			
Days	12 °C	20 °C	27 °C
	Mean ± SD	Mean ± SD	Mean ± SD
30	97.5 ± 4.33 A	97.5 ± 4.33 A	95 ± 8.66 A
60	92.5 ± 8.29 A	85 ± 5 A	77.5 ± 4.33 A
90	87.5 ± 4.33 A	72.5 ± 8.29 AB	62.5 ± 8.29 B
120	80 ± 7.07 A	55 ± 5 B	45 ± 8.66 B
150	72.5 ± 8.29 A	N/A	N/A
180	67.5 ± 9.57 A	N/A	N/A

## Data Availability

All data analyzed in this study are included in this article.
